# Ultra-Fast Ion Mobility Spectrometer for High-Throughput Chromatography

**DOI:** 10.1021/acs.analchem.3c03935

**Published:** 2023-11-13

**Authors:** Christian Thoben, Florian Schlottmann, Tim Kobelt, Alexander Nitschke, Gian-Luca Gloeden, Cameron N. Naylor, Ansgar T. Kirk, Stefan Zimmermann

**Affiliations:** Institute of Electrical Engineering and Measurement Technology, Department of Sensors and Measurement Technology, Leibniz University Hannover, Appelstraße 9A, 30167 Hannover, Germany

## Abstract

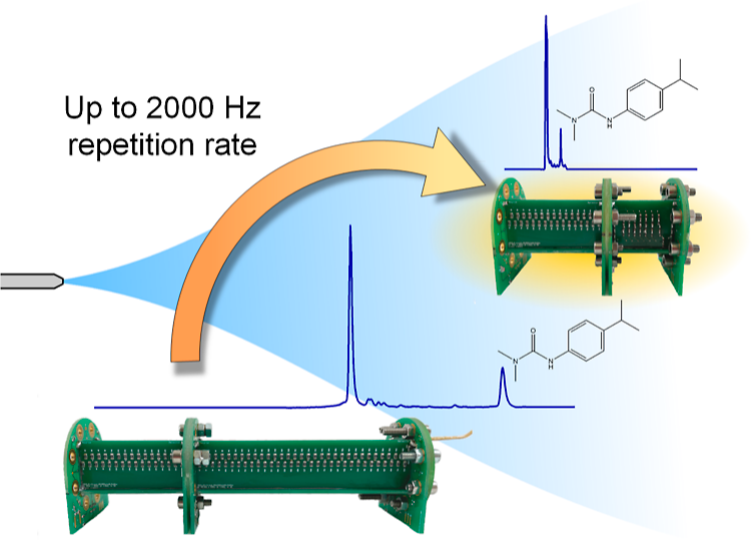

Fast chromatography systems especially developed for high sample throughput applications require sensitive detectors with a high repetition rate. These high throughput techniques, including various chip-based microfluidic designs, often benefit from detectors providing subsequent separation in another dimension, such as mass spectrometry or ion mobility spectrometry (IMS), giving additional information about the analytes or monitoring reaction kinetics. However, subsequent separation is required at a high repetition rate. Here, we therefore present an ultra-fast drift tube IMS operating at ambient pressure. Short drift times while maintaining high resolving power are reached by several key instrumental design features: short length of the drift tube, resistor network of the drift tube, tristate ion shutter, and improved data acquisition electronics. With these design improvements, even slow ions with a reduced mobility of just 0.94 cm^2^/(V s) have a drift time below 1.6 ms. Such short drift times allow for a significantly increased repetition rate of 600 Hz compared with previously reported values. To further reduce drift times and thus increase the repetition rate, helium can be used as the drift gas, which allows repetition rates of up to 2 kHz. Finally, these significant improvements enable IMS to be used as a detector following ultra-fast separation including chip-based chromatographic systems or droplet microfluidic applications requiring high repetition rates.

## Introduction

Chromatography remains a popular and powerful analytical technique due to its ability to separate even highly complex samples. Coupling any type of chromatography to an additional separation technique, such as mass spectrometry (MS) adds additional information about the analytes in the sample. For this reason, liquid chromatography (LC) coupled to MS is a well-utilized technique in various ‘omics applications such as proteomics and metabolomics.^1−3^ In addition to LC–MS, MS is coupled to gas chromatography (GC) and supercritical fluid chromatography (SFC). GC–MS is well suited to the analysis of volatile compounds including the headspace of samples, such as foodstuffs,^4^ drugs,^5^ and forensic samples.^6^ SFC-MS is able to separate nonpolar compounds, such as lipids, and perform separations faster than LC due to its supercritical fluid mobile phase.^7−9^

For all chromatographic separation techniques, challenges arise when coupling a detector that adds a second separation dimension. One of the most important considerations is the repetition rate of this second dimension as it is simultaneously the sampling rate of the first dimension and must therefore be high enough to ensure sampling a fully Gaussian chromatographic peak.^10^ Shortening the measurement time of the second dimension and thus increasing the sampling rate are therefore a strategy to increase the number of sample points across one peak of the first dimension. Miniaturization of analytical systems is one solution that often results in reduced measurement times in the seconds dimenson.^11^ However, faster chromatographic systems, including microfluidic systems, are under development that further increase sample throughput already reaching up to 500 Hz repetition rate of droplet generation.^12−14^ Such systems require even higher sampling rates to prevent timing mismatches.

Under some circumstances, ion mobility spectrometry (IMS) can serve as an alternative to mass spectrometry for additional orthogonal separation after chromatography. Ion mobility spectrometers have become a widely used instrument in many analytical^15−17^ and bioanalytical applications.^18−20^ Although the origin of IMS was gas-phase analysis,^21^ IMS has been successfully coupled to liquid-phase ionization techniques including electrospray ionization (ESI) and atmospheric pressure chemical ionization (APCI) for monitoring of chemical and biochemical reactions^22−24^ or the analysis of aqueous samples.^25−28^ Most IMS designs operate at ambient pressure, making the IMS highly portable.

Similar to mass spectrometers, IMS provides an additional benefit: another separation dimension based on the collision cross section corresponding to the measured ion mobility recorded at low-field conditions. The specific ion mobility can contribute to compound identification by ion mobility matching. Namely, an IMS separates ions via an electric field as they collide with the neutral drift gas. The drift time *t*_D_ of the ion depends on the mobility of the ion *K*, the applied electric field strength *E*_D_, and the length of the drift region *L*_D_, as described in eq 1.^21^ Consequently, the strength of this electric field and the length of the drift tube are the two most important parameters in reducing the drift time of the ions and enabling the highest possible repetition rate.
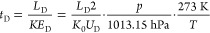
1

When trying to minimize drift times and thus maximize the repetition rate of the second dimension, physical restrictions exist. The length of the drift tube cannot be infinitely reduced, and the electric drift field cannot be increased infinitely. To bound the restrictions of decreasing drift times with optimum performance, other common IMS metrics can serve as a helpful guide. One such commonly used benchmark for the performance of an IMS is the resolving power, which is defined according to eq 2.^21,29^
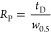
2

Resolving powers of over 80 are considered to be high-resolution IMS.^30,31^ While resolving power does not explicitly define separation capacity, in linear IMS as discussed here, it is proportional to the metric of resolution, or how well two peaks are resolved.^32^ Thus, maximizing the resolving power is a common goal in IMS instrumental design. Especially in the cases where ion drift times need to be reduced, minimizing the initial ion packet width and the amplifier width (the peak width added by the amplifier) is the primary way to increase the resolving power. In this context, the choice of the ion shutter is decisive for the reduction of the initial ion packet width. In the present work, we use a tristate ion shutter which exhibits no ion discrimination of less mobile ions even for shortest shutter opening times.^33−36^ For most other ion shutters, ion discrimination creates a limit for the lowest initial ion packet width that is still practically useable due to increasing loss of sensitivity with decreasing shutter opening times.

Here, we present the culmination of the design considerations mentioned above into an ultra-fast, drift tube ion mobility spectrometer operated at ambient pressure. By reducing the drift region, increasing the voltage, minimizing the initial ion packet width, and utilizing fast data acquisition electronics, ion drift times are significantly reduced while maintaining high resolving power. Short drift times allows for preservation of high sampling frequency when any chromatographic separation or microfluidic technique is coupled prior to the IMS. The previous maximum repetition rate reported in the literature for ambient pressure IMS was, to our best knowledge, below 50 Hz,^37^ but after our optimizations, we are able to achieve 600 Hz with purified air as drift gas and even 2 kHz with helium as drift gas. The performance of the IMS is characterized using ions generated from an ESI source; however, other ion sources such as APCI sources can be used, allowing for high flexibility in coupling chromatography systems. Although the work presented here is a preliminary effort, the results are promising for future applications, where fast data acquisition following separation is required.

## Experimental Section

### Theoretical Considerations for Achieving High Electric Field Strength in IMS

For IMS to be operated at high drift voltages, or at high electric field strengths respectively, at least two effects must be considered: (1) avoiding dielectric breakdown between the drift ring electrodes inside the IMS and (2) the resistor network used to set the potentials of the ring electrode. Schlottmann et al.^38^ discuss the first point. Although this publication deals with an IMS operated at absolute pressures of 20–60 hPa and high reduced electric field strengths of up to 120 Td,^38−40^ named high kinetic energy ion mobility spectrometer (HiKE-IMS), the considerations regarding breakdown between ring electrodes are easily transferable to any other IMS. Schlottmann et al.^38^ discuss using the Paschen curve to avoid breakdown at high reduced electric field strength between two adjacent electrodes. In summary, a high number of electrodes with the highest possible distance between two adjacent electrodes is the best method to operate below the minimum of the Paschen curve. Similar to the design from Schlottmann et al.,^38^ we use a printed circuit board (PCB) drift tube IMS to handle the required high electric fields between the electrodes to reach short drift times.

The second point of consideration is the resistor network, which must also withstand the high drift voltages applied. Therefore, it is important to consider each single resistor between the ring electrodes or grid electrodes (tristate ion shutter and aperture grid electrodes) and to ensure that neither its nominal voltage nor its nominal power dissipation is exceeded. Here, only surface-mount device (SMD) resistors are used as these can be placed directly onto the PCB-IMS and are available with small footprints. In our PCB-IMS design originally for the HiKE-IMS, high voltage resistance chip resistors from ROHM Semiconductors KTR series are used as these have high limiting voltage of 400 V (KTR10, size 0805 in imperial units) and even higher maximum overload voltage of 800 V (KTR10). Alternatively, resistors with a smaller footprint are available in this series; these also have a relatively high limiting voltage of 350 V (KTR03, size 0603 in imperial units) and a higher maximum overload voltage of 500 V (KTR03). Furthermore, the space on the outside of the PCB-IMS is limited where resistors are located. Hence, resistors with a larger footprint than the two types described above are not favored.

Voltages applied to resistor networks will inevitably lead to power dissipation in the resistors, which can lead to unwanted self-heating of PCB-IMS from energy conversion. However, unwanted heating can be minimized by a few simple considerations. Power dissipation, *P*, is given as the square of the voltage, *U*, divided by the resistance, *R*: *P* = *U*^2^/*R*. Thus, to minimize power dissipation, the resistance value of the resistors needs to be sufficiently high when high voltages are applied. Both the resistors mentioned above (KTR03 and KTR10) have the same maximum resistance (10 MΩ), but it is easier to place KTR03 resistors instead of KTR10 due to their smaller size. KTR03 resistors have a footprint roughly half as large as KTR10 resistors (KTR03:1.28 mm^2^ vs KTR10:2.5 mm^2^). Additionally, placing two KTR03 in series between two ring electrodes halves the power dissipation over the entire drift region and even reduces the power dissipation to a quarter for the single resistor, resulting in significantly decreased self-heating. Due to the finite space between each electrode, it is easier to place more of the smaller resistors in series than the larger ones. By placing more resistors in series, the voltage that can be applied between two electrodes without overloading the resistors nearly doubles, from 400 V (one KTR10) to 700 V (two KTR03). By placing the KTR03 resistors in series for our PCB-IMS, power dissipation by the resistor network is less than 0.5 W for a 34 mm long drift region when a drift voltage of 7 kV is applied.

### Data Acquisition and Pulse Generation

To achieve isolated data acquisition with data rates of up to 250 kS/s, new data acquisition and pulse generation electronics were developed based on a hybrid solution of field-programmable gate array (FPGA) and microcontroller. The isolated serial peripheral interface (SPI) connection of the analog-to-digital converter (ADC) via optical fibers is based on the work of Lippmann et al.^41^

This new data acquisition electronics is based on a Xilinx Zynq Z7020, which has two ARM Cortex-A9 cores and a programmable logic (FPGA). These two components can quickly exchange data via an AXI-bus (Advanced eXtensible Interface). The FPGA runs at 100 MHz which allows logic pulses with a resolution of 10 ns. The FPGA takes care of synchronously generating the pulses needed for IMS and reading out the ADC via the isolated SPI connection. The incoming spectra are then stored in the 512 MB (DDR3) random access memory in the form of a ring buffer. This allows the data to be buffered until the spectrum has been retrieved from the computer via TCP/IP. This ensures that each spectrum arrives in the correct order in the measurement software and can be assigned to the respective event, e.g., an eluting peak from the chromatograph or a droplet from a microfluidic platform. The microcontroller configures the FPGA and handles communication via TCP/IP with the measurement software. It also manages communication with all system components.

### Instrumental

**Figure 1:**
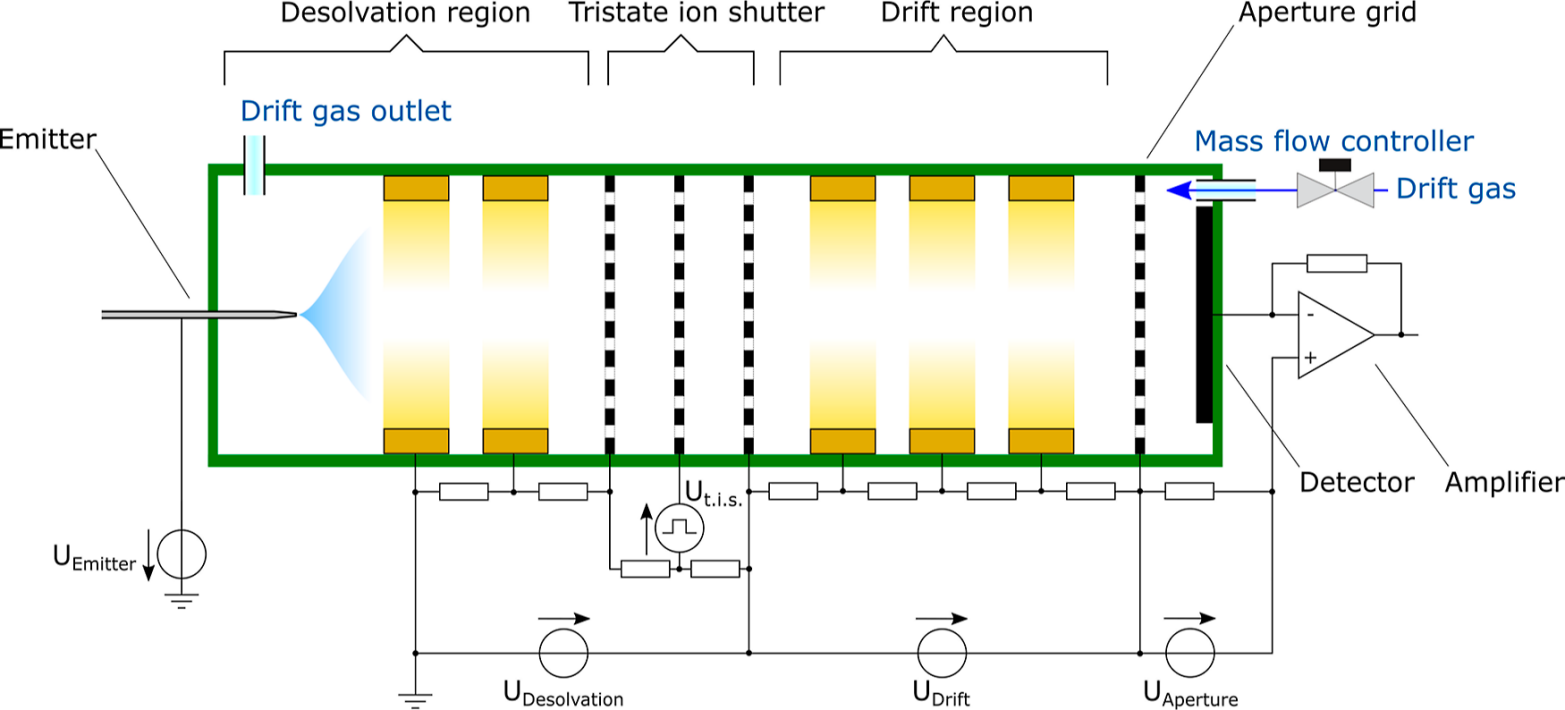
Schematic of the ESI-PCB-IMS with the voltages for the electrospray emitter, the desolvation region, the drift region, and aperture grid, as well as the pulsed voltage at the ion gate. Also shown is the controlled mass flow of the drift gas with the drift gas outlet at the beginning of the desolvation region.

In this work, a self-designed and self-constructed compact ESI-PCB-IMS with a rectangular shape of 20 mm × 20 mm, and a drift tube length of 34 mm with 22 electrodes with a center distance of 1.5 mm and a gap of 0.5 mm is used, the detailed description of the setup can be found elsewhere.^36,38^ The ions are generated by an electrospray ion source consisting of a quartz silica emitter (FossilIonTech, MS-Wil, Aarle-Rixtel, The Netherlands) with an inner tip diameter of 50 μm and a desolvation region of 50 mm length. The ion source operated at a flow rate of 0.5 μL/min. The ESI voltage of 2.7 to 3 kV is applied between the emitter and the grounded first ring of the inlet of the desolvation region. Therefore, the detector and the amplifier as well as the ADC are at a high potential of up to 20 kV. This configuration avoids a rather high electrical potential of 23 kV at the emitter and allows for easy handling of the liquid. The voltages across the desolvation region and the drift region are supplied by two self-designed and self-constructed isolated 10 kV power supplies. A self-designed and self-constructed amplifier with low noise is used as a transimpedance amplifier.^42^ A self-designed and self-constructed isolated DC power supply with 50 kV isolation and a high overall efficiency of 82.5% at 55 W is used to supply the electronics.^43^Table 1 gives an overview of the relevant operating parameters of the ESI-PCB-IMS, and an instrumental diagram is illustrated in Figure 1. An example showing how the tristate ion shutter voltage sequence can be realized with commercial components and which commercial amplifiers can be used is described in the Supporting Information.

**Table 1:** Ultra-Fast IMS Operating Parameters

parameter	value	parameter	value
length of drift region	34 mm	drift field strength	28.5–214 V/mm
length of desolvation region	50 mm	desolvation field strength	28.5–214 V/mm
emitter-to-ring voltage	2.7–3 kV	liquid flow to ESI	0.5 μL/min
drift gas flow rate	250 mL/min	emitter diameter	50 μm
drift gas dew point	–85°C	drift region temperature	22–25°C
drift gas	purified air or helium	desolvation region temperature	25–25°C
pressure (absolute)	1000–1029 hPa		

### Chemicals

LC–MS grade water and methanol (MeOH) were used as solvents and were purchased from Altmann Analytik GmbH & Co. KG, Germany. The herbicides isoproturon (analytical standard) and pyrimethanil (analytical standard) as well as the instrument standards tetraethylammonium iodide (TEAI) (ACS reagent), tetrapropylammonium iodide (TPAI) (ACS reagent), tetrabutylammonium iodide (TBAI) (ACS reagent), and tetrahexylammonium bromide (THAB) (ACS reagent) were purchased from Sigma-Aldrich Chemie GmbH, Germany. The test solution contains 5 μM each tetraalkylammonium halide in methanol. The herbicides were prepared at a concentration of 5 mg/L in 20:80 water/methanol.

## Results and Discussion

The aim of this work is to realize a drift tube ion mobility spectrometer operated at ambient pressure and with drift times as low as possible in order to use IMS as a detector for ultra-fast separation techniques, which require high detector repetition rates to resolve any signal peak of the first separation dimension. As shown in eq 1, a reduction of the drift time can be achieved by reducing the drift length of the IMS. With the definition of the applied electric field strength *E*_D_ = *U*_D_/*L*_D_, it follows that the drift time scales with the square of the drift length at constant drift voltage *U*_D_. Therefore, a drift length of only 34 mm is used in this work, which corresponds to 44% of the length of our previously used ESI-IMS systems. This means that drift times can be expected to be shorter by a factor of 5.29, and with appropriate data acquisition electronics, the repetition rate can also be improved by this factor.

### Sweep Drift Voltage

**Figure 2:**
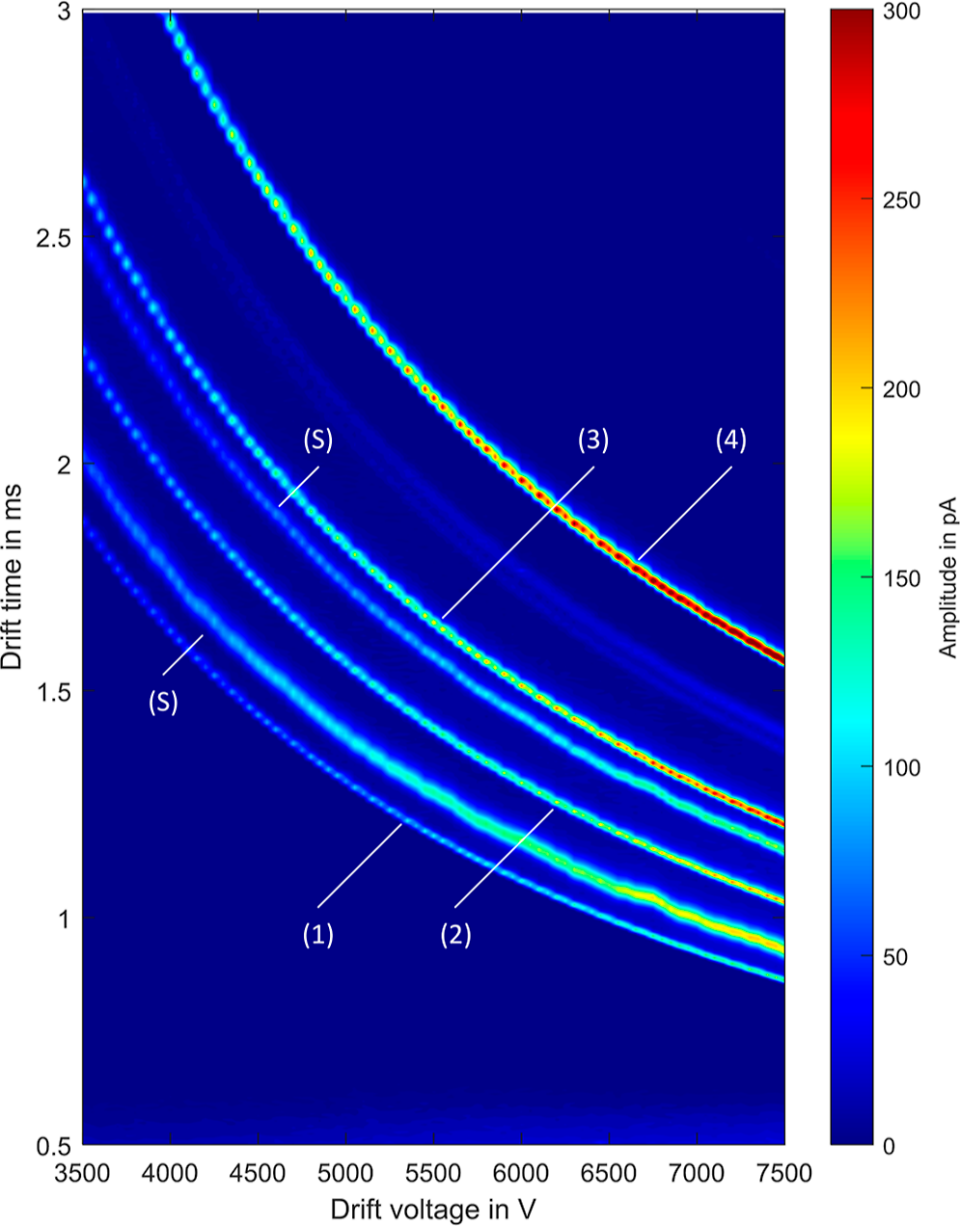
2D plot of drift times versus drift voltage for the four instrument standards TEAI (1), TPAI (2), TBAI (3), and THAB (4) in methanol used as solvent (S).

In addition, the drift time can be reduced by increasing the drift voltage. Therefore, as a first step, the drift voltage is varied from 3500 to 7500 V, and four instrument standards are used as analytes. The results are shown in Figure 2, where the drift time is plotted against the drift voltage. As expected, the drift time can be significantly reduced in this way. However, further increasing the drift voltage would result in an arc breakdown in the drift tube. At a drift voltage of 7500 V, a drift time of less than 1.6 ms can be reached for the tetrahexylammonium bromide, which has a low reduced ion mobility of *K*_0_ = 0.94 cm^2^/(V s). Therefore, a repetition rate of 600 Hz is already possible even with such slow ions present.

Theoretically, it would also be possible to shorten the drift time by reducing the pressure or increasing the temperature within the drift tube. However, increasing the temperature by 30 °C reduces the drift time by only 10%, as can be seen from eq 1. Reducing the pressure, in turn, increases the instrumental effort to implement coupling to most separation techniques, especially when ionization sources are used that operate at ambient pressure. Thus, the appropriate way to minimize the drift time is to reduce the drift length and increase the drift voltage. In order to achieve the highest possible resolving power at reduced drift length and increased drift voltage, a reduction of the initial ion packet width by optimizing the ion shutter is a suitable way.

### Optimized Resolving Power

#### Closing Field

**Figure 3:**
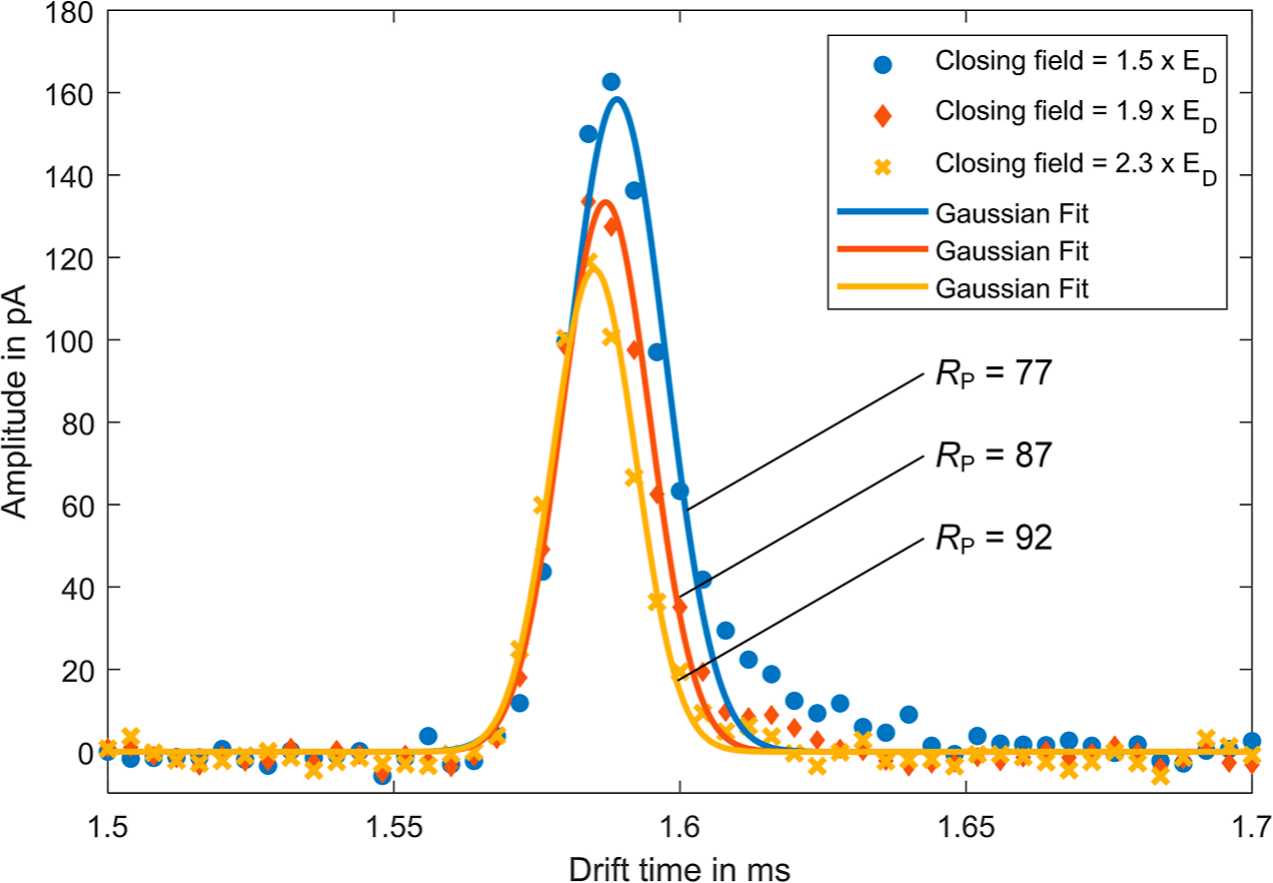
THAB peaks (extracted from the ion mobility spectra when electrospraying the four instrument standards TEAI, TPAI, TBAI, and THAB dissolved in methanol) at a drift field strength *E*_D_ = 214 V/mm for closing fields of 1.5*E*_D_ (data points: blue circles, Gaussian fit: blue line), 1.9*E*_D_ (data points: red diamond, Gaussian fit: red line), and 2.3*E*_D_ (data points: yellow crosses, Gaussian fit: yellow line) of the tristate ion shutter with the corresponding resolving powers.

Especially for the short drift time targeted, sharp and symmetric peaks are required for a high separation performance. A recurring problem in IMS is tailing of the peaks, e.g., due to field inhomogeneities when closing the ion shutter. This leads to deformation of the ion packet already at the beginning of the drift region. With the tristate ion shutter used, the initial ion packet can additionally be affected by the electric field closing the ion shutter. If the closing field is increased, the peak amplitude decreases due to increased ion loss at the last shutter grid, but at the same time, the resolving power increases due to the additional compression of the ion packet. The reduced tailing when increasing the closing field of the tristate ion shutter is shown in Figure 3 for the THAB peak as an example. A higher closing electric field strength at the ion shutter leads to a decrease in the peak amplitude, but at the same time the resolving power increases from *R*_P_ = 77 at a closing field of 1.5*E*_D_ to *R*_P_ = 92 at a closing field of 2.3*E*_D_. In order to obtain the highest possible resolving power and thus good peak resolution in the ion mobility dimension, even with the short drift tube selected, a closing field of 2.3*E*_D_ is used in the following.

#### Shutter Opening Time

**Figure 4:**
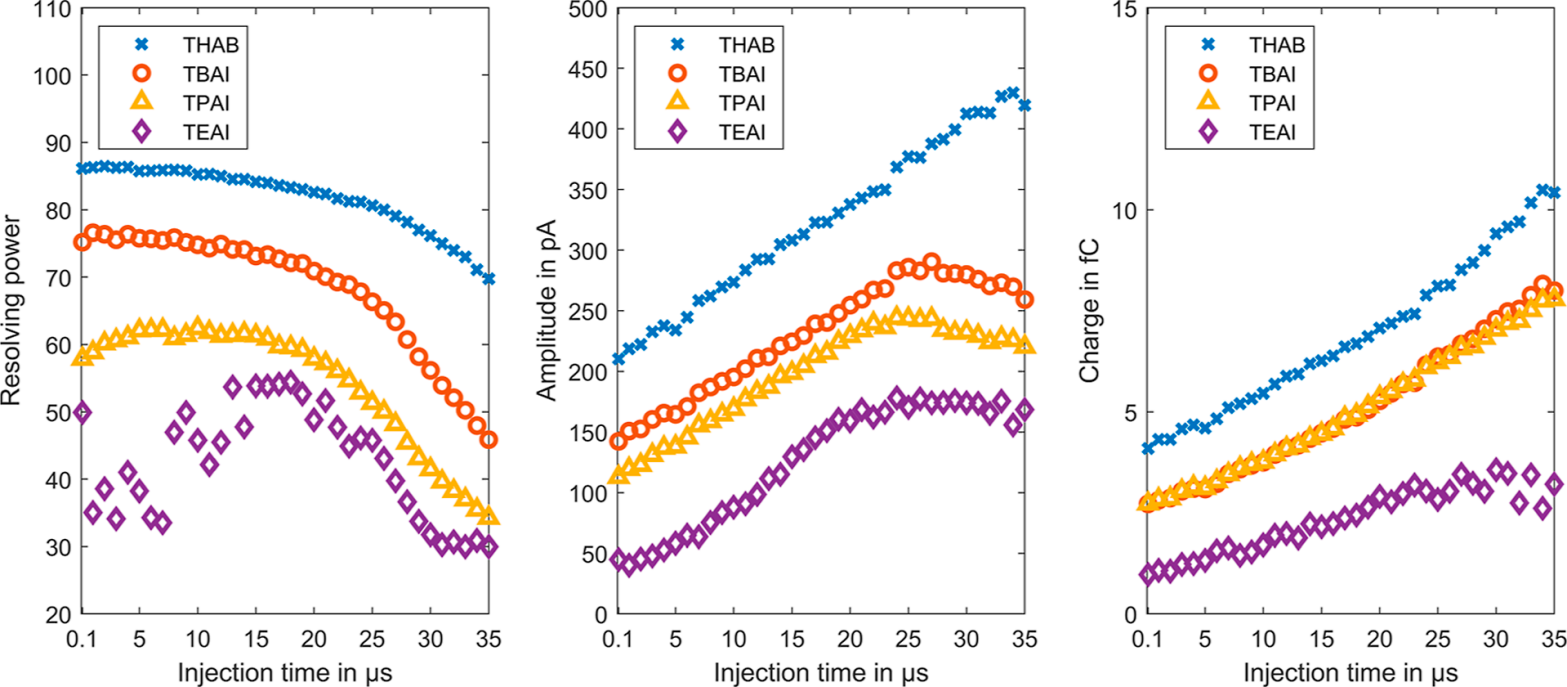
Resolving power (left), amplitude (middle), and the integral, respectively, charge (right) of the peaks for TEAI (violet diamond), TPAI (yellow triangles), TBAI (red circles), and THAB (blue crosses) for different injection times from 0.1 to 35 μs.

Besides the tailing, especially the opening time of the ion shutter has an influence on the amplitude, the peak integral, and the resolving power. Figure 4 shows the resolving power, the peak amplitude, and the integral respectively charge of the analyte peak over the ion shutter opening time for each of the four instrument standards. From these measurements, it is clear that the lowest possible shutter opening time should be selected for highest resolving power. However, as diffusion is still the main contributor to the peak width, up to an opening time of 25 μs, the resolving power degrades only slightly with a simultaneous increase in the peak amplitude and peak integral. At opening times longer than 25 μs, the resolving power decreases significantly as the ion shutter opening time dominates the peak width. An increase of the peak amplitude is no longer achieved, only the peak integral still increases with the opening time due to the broadening of the peaks. According to the amplitude, the sensitivity is approximately halved when the ion shutter opening time is reduced from 25 to 5 μs. Thus, depending on the application, the shutter opening time should be selected between 5 and 25 μs.

At an opening time of just 0.1 μs, the ions only have a spatial width of a few μm, depending on their ion mobility, theoretically preventing them from passing the middle grid used for the ion shutter having a width of 100 μm. However, the substances can still be detected at this short opening time. The explanation lies in the field lines, which extend beyond the middle grid in the first closed state of the ion shutter. When the ion shutter is briefly opened and switched to the second closed state, any ions located between the middle and third grids due to these field lines are released into the drift region. This effect depends on the exact ion shutter geometry and has therefore been observed in some tristate ion shutters^44,45^ but not others.^33,36^ Here, this nonideality is not relevant for the application as an ultrafast detector. An illustrative animation for an ion shutter “opening” time of 0 μs can be found in the Supporting Information.

The trade-off between amplitude and resolving power can also be illustrated by the two herbicides isoproturon and pyrimethanil, as shown in Figure S2.

#### Transimpedance Amplifier

Besides the obvious settings at the ion shutter, the transimpedance amplifier also has a significant influence on the resolving power and the peak amplitude,^46,47^ especially if the amplifier has to amplify the very narrow peaks with half widths of only 16 μs. In the following, three different transimpedance amplifier parameter sets with different gains and different bandwidths are compared with each other using the four instrument standards. The parameters of the amplifiers are listed in Table 2. First, Figure S3 shows that excessive amplifier bandwidth does not provide any improvement with respect to resolving power, while low bandwidth broadens the peak significantly.

**Table 2:** Amplifiers’ Parameters

name	bandwidth (kHz)	amplifier width (μs)^46^	gain (GΩ)
parameter set 1	29	10.9	4.790
parameter set 2	261	1.3	0.425
parameter set 3	105	3.0	2.520

However, to optimize both resolving power and signal-to-noise ratio (SNR), further aspects have to be considered as amplifiers with higher bandwidth also add more noise to the spectrum. It can be shown that for a given initial width of the ion packet, the optimum SNR is achieved when the initial width of the ion packet and the width added by the amplifier due to its low pass characteristic are of the same value.^46,47^ Descriptively, an amplifier being too fast adds too much noise to the spectrum, decreasing SNR, and an amplifier being too slow leads to excessive peak broadening and reduced peak amplitude, also decreasing SNR. In the following, an initial ion packet width of 5 μs is chosen for this consideration. It follows that when low-pass filtering the spectra to set the desired bandwidth, the cutoff frequency of the low-pass filter should be set to 57 kHz so that the initial width of the ion packet and the width added by the amplifier are approximately the same. The conversion of the measurement signal into its frequency components via a discrete or fast Fourier transform reveals that the useful signal is indeed 95% concentrated below 57 kHz. Earlier considerations about a simple configuration of a low-pass filter to achieve denoising and an improved SNR lead to similar results.^48^

**Figure 5:**
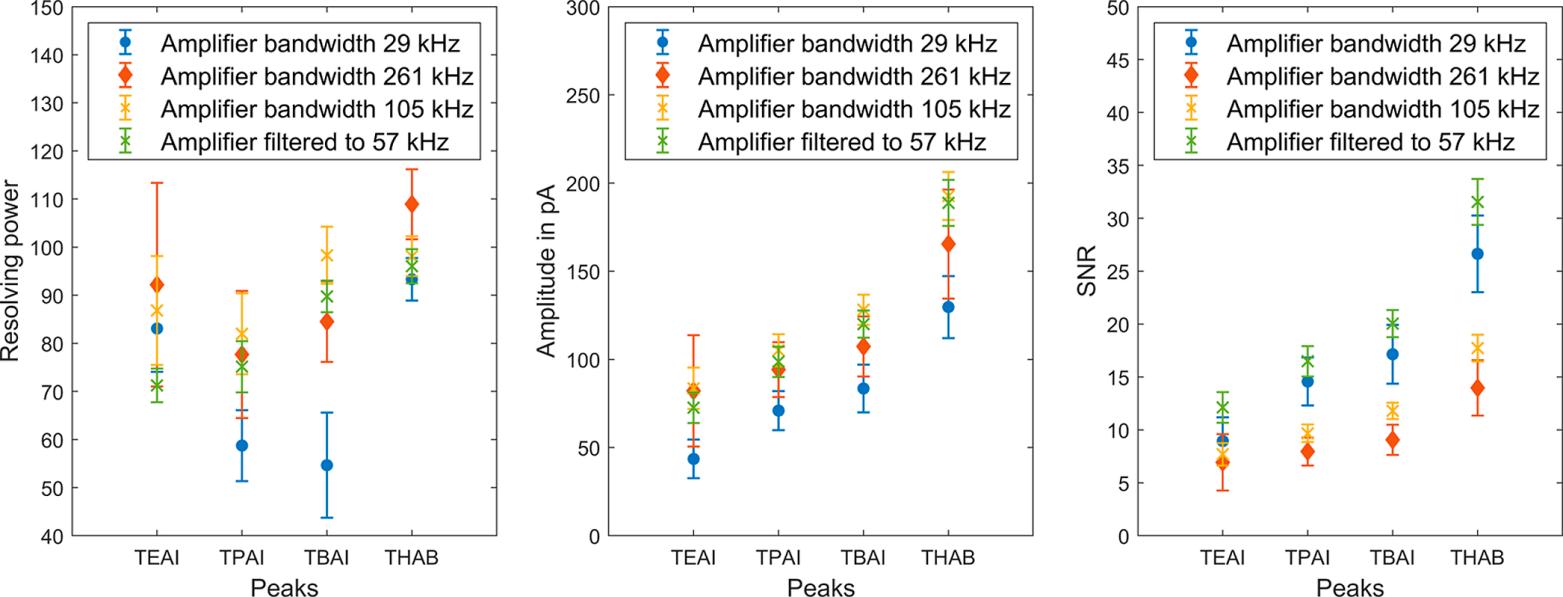
Resolving power, amplitude in pA, and SNR of the TEAI, TPAI, TBAI and THAB peaks recorded with different transimpedance amplifiers with a bandwidth of 29 kHz (blue rings), 261 kHz (red circles), and 105 kHz (yellow crosses) without averaging, in addition the low-pass filtered data of the 105 kHz amplifier with the cutoff frequency of 57 kHz (green crosses).

As shown in Figure 5, the different transimpedance amplifiers with different gains and different bandwidths are compared with each other using the four instrument standards without averaging the spectra. If the amplitude is considered, the two amplifiers with higher bandwidths outperform the amplifier with the lowest bandwidth of 29 kHz, even though this amplifier has the highest gain. However, in the raw data, the bandwidth dominates the SNR, but with low-pass filtering to 57 kHz, the values for the 105 kHz bandwidth amplifier are improved in this respect.

Overall, the amplifier with the highest gain and smallest bandwidth is not suited since it does not follow the measurement signal sufficiently, leading to peak broadening of the analyte peaks and thus to reduced peak amplitudes. If the application requires the highest possible repetition rate without averaging, an amplifier with sufficient but not too high bandwidth should be used as this offers optimum resolving power and best obtainable SNR.^46,47^

### Drift Gas Contribution

For further reducing the drift time, there is still the possibility to select a suitable drift gas.^49−51^ A promising method is using pure helium as a drift gas or adding helium to the drift gas. This increases ion mobility depending on the helium content and thus reduces drift time so that a faster repetition rate becomes possible.

**Figure 6:**
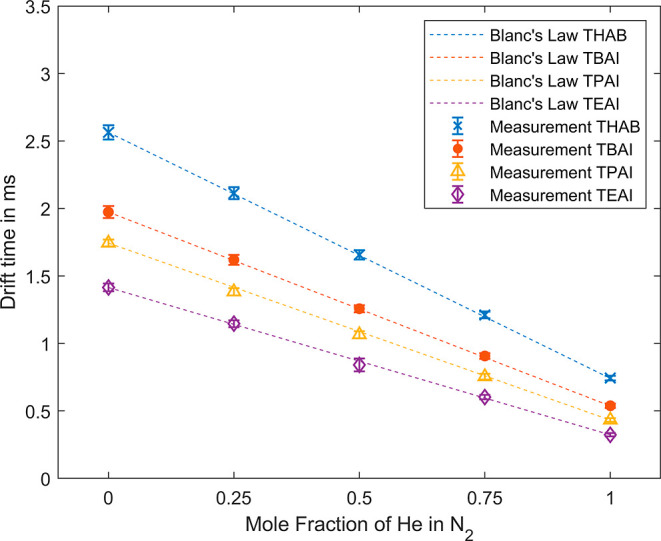
Drift time of the four instrument standards, TEAI (purple diamond), TPAI (yellow triangle), TBAI (red circle), and THAB (blue cross) versus mole fraction of helium in nitrogen of the drift gas. The error bars represent the base width of the corresponding peaks. The dashed lines show the calculations of the drift times via Blanc’s law from the data of 100% nitrogen and 100% helium as drift gas.

The application of Blanc’s law^52^ allows the prediction of ion mobility values for drift gas mixtures. This requires two known ion mobilities obtained from different drift gases, e.g., each drift gas component of the drift gas mixture. For this purpose, either literature values are used or, as shown here, the ion mobilities in two different drift gases are measured. To calculate the ion mobilities using Blanc’s law, the instrument standards were measured in pure nitrogen and pure helium at a drift voltage of 5 kV. The measured reduced ion mobilities of the instrument standards are listed in Table 3. The Blanc’s law predictions are shown in Figure 6 as dashed lines for the respective standards. For verification, the ion mobilities were measured at three different mole fractions of the drift gas mixture, which agree very well with the predictions of Blanc’s law. Thus, for all mixtures, the desired drift time can be predicted and adjusted by the mole fraction of the drift gas. As the drift times and the half-widths decrease with increasing helium content, the resolution between the peaks remains almost the same.

**Table 3:** Reduced Mobility Values of Each of the Analytes in the Respective Drift Gas

	*K*_0_ in nitrogen (cm^2^ V^–^^1^ s^–^^1^)	*K*_0_ in helium (cm^2^ V^–^^1^ s^–^^1^)
TEAI	1.70	7.43
TPAI	1.39	5.55
TBAI	1.22	4.47
THAB	0.94	3.24

**Figure 7:**
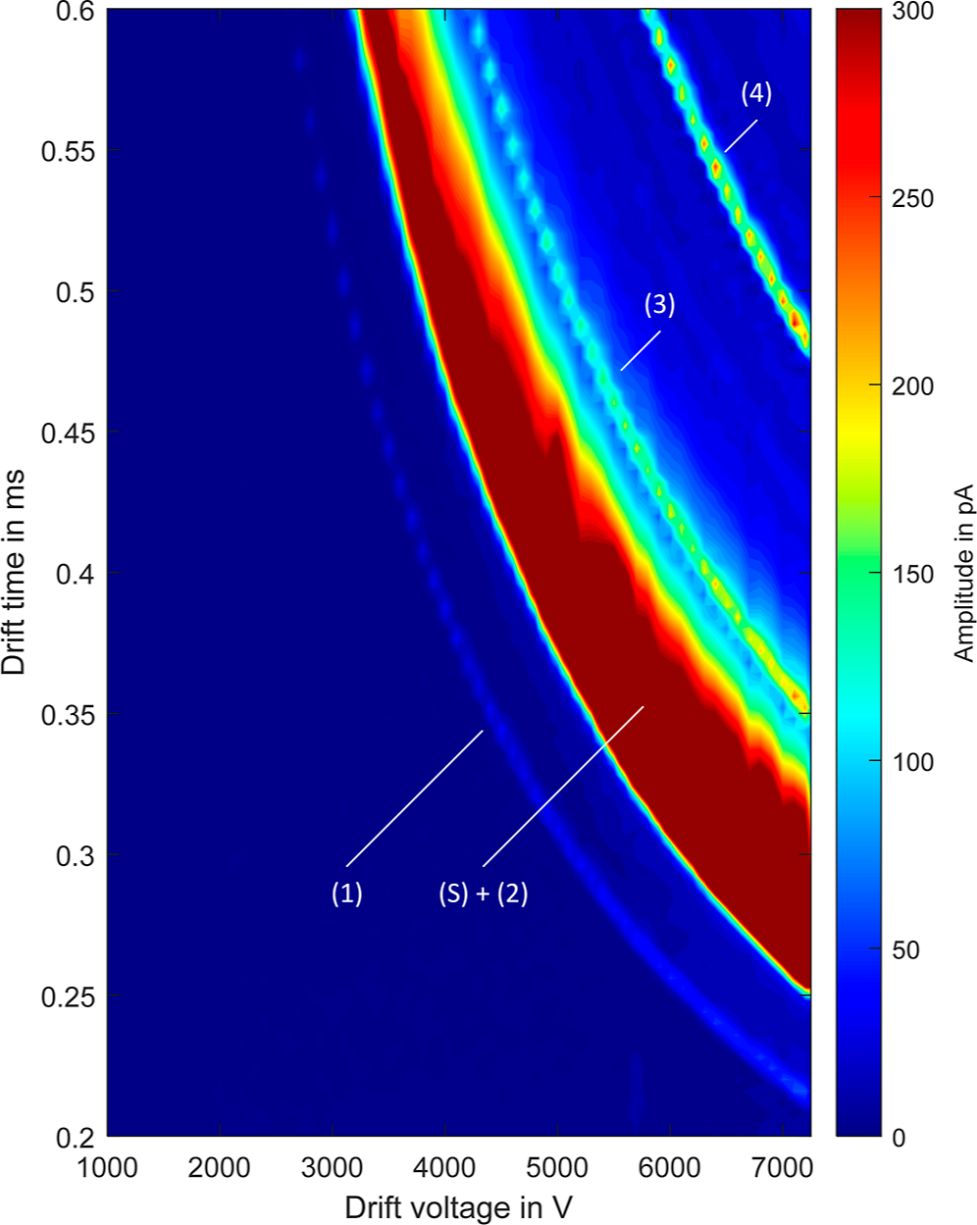
2D plot of drift times versus drift voltage of the instrument standards TEAI (1), TPAI (2), TBAI (3), and THAB (4) dissolved in methanol (S) and measured in pure helium as drift gas. Within the methanol peak (S), the TPAI peak (2) is located since the number of collisions in the desolvation region is not sufficient to decluster the solvent ions.

Of course, the shortest drift times are achieved in pure helium. For this reason, the drift voltage was again varied stepwise from 1000 to 7500 V with pure helium as drift gas. Above 7000 V drift voltage, THAB has a drift time of less than 0.5 ms, so that a repetition rate of up to 2 kHz becomes possible, as shown in Figure 7. It is also noticeable that the solvent can no longer be sufficiently declustered and clearly separated from the TPAI. However, all substances that range between the ion mobilities of TBAI and THAB can be separated and detected with the presented system with a repetition rate of 2 kHz. Such a high repetition rate even allows for averaging of ion mobility spectra despite ultra-fast separation in the first dimension or the fast succession of droplets in droplet microfluidics.

## Conclusions

By reducing the drift tube length and increasing the drift field, an ion’s drift time can be significantly reduced. If the ion shutter opening time is reduced at the same time and the amplifier bandwidth is matched to the ion shutter opening time, high separation performance is possible compared to IMS with longer drift tubes. Specifically, with a drift tube length of just 34 mm, high drift voltage of 7500 V, and ions with *K*_0_ ≥ 0.94 cm^2^/(V s) in nitrogen, short drift times below 1.6 ms are reached allowing for high repetition rates of at least 600 Hz. With improved data acquisition electronics, peak fidelity is maintained even at ultra-short drift times. Using the tristate ion shutter, short opening times in the μs range allow for high resolving power of *R*_p_ = 90 even with the relatively short drift tube of 34 mm. By changing the drift gas from nitrogen to helium or using helium/nitrogen mixtures, the drift times can be reduced even further. The inverse ion mobility decreases linearly as a function of the helium content in the drift gas, resulting in shorter drift times and, thus, even faster repetition rates. Specifically, in pure helium, THAB with *K*_0_ = 0.94 cm^2^/(V s) has a drift time of less than 0.5 ms, resulting in a repetition rate of 2 kHz. All these instrument design features enable an ultra-fast IMS that can be used as a detector with sufficient repetition rate for ultra-fast separation techniques while providing another dimension of separation. In particular, this instrument is beneficial for droplet microfluidics because the sample rate is significantly higher than what has been reported for previous IMS coupled to microfluidics.^13^ Additionally, the ultrafast IMS can be coupled to ultra-fast chromatography including miniaturized GC, LC, or SFC. These couplings allow for further development of future ultra-fast chromatography instruments and subsequent developments in prototyping applications benefiting from high throughput such as pharmaceutical development.^53^

## Supporting Information Available

Simulation of an ion injection using the tristate shutter with a shutter “opening” time of 0 μs (AVI)
Implementation of the tristate ion shutter voltage sequence with commercial components, ion mobility spectra for different injection times from 0.1 to 25 μs and THAB peaks measured with different amplifier bandwidths (PDF)

## Supplementary Material

ac3c03935_si_001.avi

## References

[ref1] Delvaux A.; Rathahao-Paris E.; Alves S. Different ion mobility-mass spectrometry coupling techniques to promote metabolomics. Mass Spectrom. Rev. 2022, 41, 695–721. 10.1002/mas.21685. 33492707

[ref2] Gallien S.; Duriez E.; Demeure K.; Domon B. Selectivity of LC-MS/MS analysis: implication for proteomics experiments. J. Proteomics 2013, 81, 148–158. 10.1016/j.jprot.2012.11.005. 23159602

[ref3] Motoyama A.; Yates J. R. Multidimensional LC separations in shotgun proteomics. Anal. Chem. 2008, 80, 7187–7193. 10.1021/ac8013669. 18826178

[ref4] Mondello L.; Casilli A.; Tranchida P. Q.; Dugo P.; Costa R.; Festa S.; Dugo G. Comprehensive multidimensional GC for the characterization of roasted coffee beans. J. Sep. Sci. 2004, 27, 442–450. 10.1002/jssc.200301662. 15335079

[ref5] Sun M.; Bai L.; Liu D. Q. A generic approach for the determination of trace hydrazine in drug substances using in situ derivatization-headspace GC-MS. J. Pharm. Biomed. Anal. 2009, 49, 529–533. 10.1016/j.jpba.2008.11.009. 19097722

[ref6] Stambouli A.; El Bouri A.; Bouayoun T.; Bellimam M. A. Headspace-GC/MS detection of TATP traces in post-explosion debris. Forensic Sci. Int. 2004, 146, S191–S194. 10.1016/j.forsciint.2004.09.060. 15639574

[ref7] West C. Current trends in supercritical fluid chromatography. Anal. Bioanal. Chem. 2018, 410, 6441–6457. 10.1007/s00216-018-1267-4. 30051210

[ref8] Losacco G. L.; Veuthey J.-L.; Guillarme D. Supercritical fluid chromatography - Mass spectrometry: Recent evolution and current trends. TrAC, Trends Anal. Chem. 2019, 118, 731–738. 10.1016/j.trac.2019.07.005.

[ref9] Chollet C.; Boutet-Mercey S.; Laboureur L.; Rincon C.; Méjean M.; Jouhet J.; Fenaille F.; Colsch B.; Touboul D. Supercritical fluid chromatography coupled to mass spectrometry for lipidomics. J. Mass Spectrom. 2019, 54, 791–801. 10.1002/jms.4445. 31652381

[ref10] Morrison K. A.; Clowers B. H. Fundamentals and applications of incorporating chromatographic separations with ion mobility-mass spectrometry. TrAC, Trends Anal. Chem. 2019, 119, 115625 10.1016/j.trac.2019.115625.

[ref11] Piendl S. K.; Raddatz C.-R.; Hartner N. T.; Thoben C.; Warias R.; Zimmermann S.; Belder D. 2D in Seconds: Coupling of Chip-HPLC with Ion Mobility Spectrometry. Anal. Chem. 2019, 91, 7613–7620. 10.1021/acs.analchem.9b00302. 31082255

[ref12] Payne E. M.; Holland-Moritz D. A.; Sun S.; Kennedy R. T. High-throughput screening by droplet microfluidics: perspective into key challenges and future prospects. Lab Chip 2020, 20, 2247–2262. 10.1039/D0LC00347F. 32500896 PMC12183681

[ref13] Kempa E. E.; Smith C. A.; Li X.; Bellina B.; Richardson K.; Pringle S.; Galman J. L.; Turner N. J.; Barran P. E. Coupling Droplet Microfluidics with Mass Spectrometry for Ultrahigh-Throughput Analysis of Complex Mixtures up to and above 30 Hz. Anal. Chem. 2020, 92, 12605–12612. 10.1021/acs.analchem.0c02632. 32786490 PMC8009470

[ref14] Steyer D. J.; Kennedy R. T. High-Throughput Nanoelectrospray Ionization-Mass Spectrometry Analysis of Microfluidic Droplet Samples. Anal. Chem. 2019, 91, 6645–6651. 10.1021/acs.analchem.9b00571. 31033282 PMC7848793

[ref15] Zou W.-B.; Yin L.-H.; Jin S.-H. Advances in rapid drug detection technology. J. Pharm. Biomed. Anal. 2018, 147, 81–88. 10.1016/j.jpba.2017.08.016. 28844368

[ref16] Ahrens A.; Allers M.; Bock H.; Hitzemann M.; Ficks A.; Zimmermann S. Detection of Chemical Warfare Agents with a Miniaturized High-Performance Drift Tube Ion Mobility Spectrometer Using High-Energetic Photons for Ionization. Anal. Chem. 2022, 94, 15440–15447. 10.1021/acs.analchem.2c03422. 36301910 PMC9647701

[ref17] Jurado-Campos N.; Martín-Gómez A.; Saavedra D.; Arce L. Usage considerations for headspace-gas chromatography-ion mobility spectrometry as a suitable technique for qualitative analysis in a routine lab. J. Chromatogr. A 2021, 1640, 461937 10.1016/j.chroma.2021.461937. 33556680

[ref18] Jeanne Dit Fouque K.; Fernandez-Lima F. Recent advances in biological separations using trapped ion mobility spectrometry - mass spectrometry. TrAC, Trends Anal. Chem. 2019, 116, 308–315. 10.1016/j.trac.2019.04.010.

[ref19] Borsdorf H.; Baldeweg S.; Löper F.; Höhnisch M.; Petrich R.; Mayer T. The correlation of odors in the environment with ion mobility spectra patterns. Int. J. Ion Mobility Spectrom. 2015, 18, 1–7. 10.1007/s12127-014-0161-9.

[ref20] Nakhodchi S.; Alizadeh N. Rapid simultaneous determination of ketamine and midazolam in biological samples using ion mobility spectrometry combined by headspace solid-phase microextraction. J. Chromatogr. A 2021, 1658, 462609 10.1016/j.chroma.2021.462609. 34656845

[ref21] Eiceman G. A.; Karpas Z.; Hill H. H. Ion Mobility Spectrometry, 3rd edn.; CRC Press: Boca Raton, 2013.

[ref22] Zühlke M.; Sass S.; Riebe D.; Beitz T.; Löhmannsröben H.-G. Real-Time Reaction Monitoring of an Organic Multistep Reaction by Electrospray Ionization-Ion Mobility Spectrometry. ChemPlusChem 2017, 82, 1266–1273. 10.1002/cplu.201700296. 31957990

[ref23] Prüfert C.; Urban R. D.; Fischer T. G.; Villatoro J.; Riebe D.; Beitz T.; Belder D.; Zeitler K.; Löhmannsröben H.-G. In situ monitoring of photocatalyzed isomerization reactions on a microchip flow reactor by IR-MALDI ion mobility spectrometry. Anal. Bioanal. Chem. 2020, 412, 7899–7911. 10.1007/s00216-020-02923-y. 32918557 PMC7550389

[ref24] Hartner N. T.; Wink K.; Raddatz C.-R.; Thoben C.; Schirmer M.; Zimmermann S.; Belder D. Coupling Droplet Microfluidics with Ion Mobility Spectrometry for Monitoring Chemical Conversions at Nanoliter Scale. Anal. Chem. 2021, 93, 13615–13623. 10.1021/acs.analchem.1c02883. 34592821

[ref25] Thoben C.; Werres T.; Henning I.; Simon P. R.; Zimmermann S.; Schmidt T. C.; Teutenberg T. Towards a miniaturized on-site nano-high performance liquid chromatography electrospray ionization ion mobility spectrometer with online enrichment. Green Anal. Chem. 2022, 1, 100011 10.1016/j.greeac.2022.100011.

[ref26] Zühlke M.; Riebe D.; Beitz T.; Löhmannsröben H.-G.; Andreotti S.; Reinert K.; Zenichowski K.; Diener M. High-performance liquid chromatography with electrospray ionization ion mobility spectrometry: Characterization, data management, and applications. J. Sep. Sci. 2016, 39, 4756–4764. 10.1002/jssc.201600749. 27805770

[ref27] Zarejousheghani M.; Schrader S.; Möder M.; Mayer T.; Borsdorf H. Negative electrospray ionization ion mobility spectrometry combined with paper-based molecular imprinted polymer disks: A novel approach for rapid target screening of trace organic compounds in water samples. Talanta 2018, 190, 47–54. 10.1016/j.talanta.2018.07.076. 30172535

[ref28] Dwivedi P.; Matz L. M.; Atkinson D. A.; Hill H. H. Jr. Electrospray ionization-ion mobility spectrometry: a rapid analytical method for aqueous nitrate and nitrite analysis. Analyst 2004, 129, 139–144. 10.1039/b311098b. 14752557

[ref29] Siems W. F.; Wu C.; Tarver E. E.; Hill H. H. Jr.; Larsen P. R.; McMinn D. G. Measuring the Resolving Power of Ion Mobility Spectrometers. Anal. Chem. 1994, 66, 4195–4201. 10.1021/ac00095a014.

[ref30] Kirk A. T.; Bohnhorst A.; Raddatz C.-R.; Allers M.; Zimmermann S. Ultra-high-resolution ion mobility spectrometry-current instrumentation, limitations, and future developments. Anal. Bioanal. Chem. 2019, 411, 6229–6246. 10.1007/s00216-019-01807-0. 30957205

[ref31] Shvartsburg A. A.; Smith R. D. Ultrahigh-resolution differential ion mobility spectrometry using extended separation times. Anal. Chem. 2011, 83, 23–29. 10.1021/ac102689p. 21117630 PMC3012152

[ref32] Dodds J. N.; May J. C.; McLean J. A. Correlating Resolving Power, Resolution, and Collision Cross Section: Unifying Cross-Platform Assessment of Separation Efficiency in Ion Mobility Spectrometry. Anal. Chem. 2017, 89, 12176–12184. 10.1021/acs.analchem.7b02827. 29039942 PMC5744666

[ref33] Kirk A. T.; Grube D.; Kobelt T.; Wendt C.; Zimmermann S. High-Resolution High Kinetic Energy Ion Mobility Spectrometer Based on a Low-Discrimination Tristate Ion Shutter. Anal. Chem. 2018, 90, 5603–5611. 10.1021/acs.analchem.7b04586. 29624371

[ref34] Thoben C.; Raddatz C.-R.; Lippmann M.; Salehimoghaddam Z.; Zimmermann S. Electrospray ionization ion mobility spectrometer with new tristate ion gating for improved sensitivity for compounds with lower ion mobility. Talanta 2021, 233, 122579 10.1016/j.talanta.2021.122579. 34215071

[ref35] Butalewicz J. P.; Sanders J. D.; Clowers B. H.; Brodbelt J. S. Improving Ion Mobility Mass Spectrometry of Proteins through Tristate Gating and Optimization of Multiplexing Parameters. J. Am. Soc. Mass Spectrom. 2023, 34, 101–108. 10.1021/jasms.2c00274. 36469482

[ref36] Bohnhorst A.; Kirk A. T.; Zimmermann S. Toward Compact High-Performance Ion Mobility Spectrometers: Ion Gating in Ion Mobility Spectrometry. Anal. Chem. 2021, 93, 6062–6070. 10.1021/acs.analchem.0c04140. 33825452

[ref37] Gerhardt N.; Birkenmeier M.; Sanders D.; Rohn S.; Weller P. Resolution-optimized headspace gas chromatography-ion mobility spectrometry (HS-GC-IMS) for non-targeted olive oil profiling. Anal. Bioanal. Chem. 2017, 409, 3933–3942. 10.1007/s00216-017-0338-2. 28417171

[ref38] Schlottmann F.; Schaefer C.; Kirk A.; Bohnhorst A.; Zimmermann S. A High Kinetic Energy Ion Mobility Spectrometer for Operation at Higher Pressures of up to 60 mbar. J. Am. Soc. Mass Spectrom. 2023, 34, 893–904. 10.1021/jasms.2c00365. 36999893 PMC10161227

[ref39] Langejuergen J.; Allers M.; Oermann J.; Kirk A. T.; Zimmermann S. High kinetic energy ion mobility spectrometer: quantitative analysis of gas mixtures with ion mobility spectrometry. Anal. Chem. 2014, 86, 7023–7032. 10.1021/ac5011662. 24937741

[ref40] Schlottmann F.; Kirk A. T.; Allers M.; Bohnhorst A.; Zimmermann S. High Kinetic Energy Ion Mobility Spectrometry (HiKE-IMS) at 40 mbar. J. Am. Soc. Mass Spectrom. 2020, 31, 1536–1543. 10.1021/jasms.0c00098. 32432872

[ref41] Lippmann M.; Kirk A. T.; Hitzemann M.; Zimmermann S. IMS Instrumentation I: Isolated data acquisition for ion mobility spectrometers with grounded ion sources. Int. J. Ion Mobility Spectrom. 2020, 23, 69–74. 10.1007/s12127-020-00260-5.

[ref42] Cochems P.; Kirk A. T.; Zimmermann S. In-circuit-measurement of parasitic elements in high gain high bandwidth low noise transimpedance amplifiers. Rev. Sci. Instrum. 2014, 85, 124703 10.1063/1.4902854. 25554310

[ref43] Hitzemann M.; Lippmann M.; Trachte J.; Nitschke A.; Burckhardt O.; Zimmermann S. Wireless Low-Power Transfer for Galvanically Isolated High-Voltage Applications. Electronics 2022, 11, 923 10.3390/electronics11060923.

[ref44] Kwantwi-Barima P.; Reinecke T.; Clowers B. H. Increased ion throughput using tristate ion-gate multiplexing. Analyst 2019, 144, 6660–6670. 10.1039/C9AN01585J. 31595887

[ref45] Chen H.; Chen C.; Huang W.; Li M.; Xiao Y.; Jiang D.; Li H. Miniaturized Ion Mobility Spectrometer with a Dual-Compression Tristate Ion Shutter for On-Site Rapid Screening of Fentanyl Drug Mixtures. Anal. Chem. 2019, 91, 9138–9146. 10.1021/acs.analchem.9b01700. 31251030

[ref46] Kirk A. T.; Allers M.; Cochems P.; Langejuergen J.; Zimmermann S. A compact high resolution ion mobility spectrometer for fast trace gas analysis. Analyst 2013, 138, 5200–5207. 10.1039/c3an00231d. 23678483

[ref47] Kirk A. T.; Bohnhorst A.; Zimmermann S. Analytical model for the signal-to-noise-ratio of drift tube ion mobility spectrometers. TM—Tech. Mess. 2021, 88, 262–273. 10.1515/teme-2021-0013.

[ref48] Goubran R. A.; Lawrence A. H. Experimental signal analysis in ion mobility spectrometry. Int. J. Mass Spectrom. Ion Processes 1991, 104, 163–178. 10.1016/0168-1176(91)80008-B.

[ref49] Naylor C. N.; Reinecke T.; Clowers B. H. Assessing the Impact of Drift Gas Polarizability in Polyatomic Ion Mobility Experiments. Anal. Chem. 2020, 92, 4226–4234. 10.1021/acs.analchem.9b04468. 32058698

[ref50] Asbury G. R.; Hill H. H. Using Different Drift Gases To Change Separation Factors (α) in Ion Mobility Spectrometry. Anal. Chem. 2000, 72, 580–584. 10.1021/ac9908952. 10695145

[ref51] Morris C. B.; May J. C.; Leaptrot K. L.; McLean J. A. Evaluating Separation Selectivity and Collision Cross Section Measurement Reproducibility in Helium, Nitrogen, Argon, and Carbon Dioxide Drift Gases for Drift Tube Ion Mobility-Mass Spectrometry. J. Am. Soc. Mass Spectrom. 2019, 30, 1059–1068. 10.1007/s13361-019-02151-4. 30887459 PMC6520154

[ref52] Blanc A. Recherches sur les mobilités des ions dans les gaz. J. Phys. Theor. Appl. 1908, 7, 825–839. 10.1051/jphystap:019080070082501.

[ref53] Beccaria M.; Cabooter D. Current developments in LC-MS for pharmaceutical analysis. Analyst 2020, 145, 1129–1157. 10.1039/C9AN02145K. 31971527

