# Supplementary material for: Ultra-Fast Ion Mobility Spectrometer for High-Throughput Chromatography
Source: Anal Chem. 2023 Nov 13;95(46):17073–81. doi: 10.1021/acs.analchem.3c03935 (PMC10666085; doi:10.1021/acs.analchem.3c03935)
Supplement: Supplementary file 2 — ac3c03935_si_002.pdf [file ac3c03935_si_002.pdf]

Supporting Information for Ultra-fast ion mobility spectrometer for high throughput chromatography

Christian Thoben,* Florian Schlottmann, Tim Kobelt, Alexander Nitschke, Gian-Luca Gloeden, Cameron N. Naylor, Ansgar T. Kirk and Stefan Zimmermann

Leibniz University Hannover, Institute of Electrical Engineering and Measurement Technology, Department of Sensors and Measurement Technology, Appelstraße 9A, 30167 Hannover, Germany

*E-mail: thoben@geml.uni-hannover.de

Table of Contents:

Figure S1: Example schematic diagram of the setup with commercially available components for providing the voltage sequence of the center grid of the tristate ion shutter.

Figure S2: Pulse sequences of a two-channel function generator to exemplarily provide the voltage sequence of the middle grating of the tristate ion shutter.

Figure S3: Ion mobility spectra of 5 mg/l isoproturon in 80:20 MeOH:H_2O (left) and of 5 mg/l pyrimethanil in 80:20 MeOH:H_2O (right) at a drift voltage of 7500 V for different injection times from 0.1 µs to 25 µs.

Figure S4: THAB peaks using transimpedance amplifier with different bandwidth of 29 kHz, 261 kHz and 105 kHz and the corresponding resolving powers.

Video S1: Simulation of an ion injection using the tristate shutter with a shutter "opening" time of 0 µs

EXPERIMENTAL

Instrumental

In addition to the presented self-built setup, all relevant components of the setup can also be realized with commercial parts. Figure S1 shows a schematic diagram of how, for example, the voltage sequence can be provided at the center grid of the tristate ion shutter. The following or similar components can be used: a two-channel DG4062 function generator from RIGOL Technologies Co., Ltd., two HTS31-06-C high-voltage switches from Behlke Power Electronics GmbH and two ESC-2 isolated high voltage power supplies from XP Power Ltd. The pulse sequences of the two channels of the function generator are shown in Figure S2. This results in the following voltages at the middle grid of the tristate ion shutters: If only the lower switch is closed, the grid is at the low reference potential, if both switches are open, the opening voltage of one power supply is applied to the grid. If only the upper switch is closed, the middle grid is at a higher potential to realize the second closing state of the tristate sequence. Furthermore, all shown experiments concerning the transimpedance amplifier can also be performed with commercial amplifiers, such as the LCA-20K-200M, LCA-100K-50M and LCA-200K-20M or DLPCA-200 from FEMTO Messtechnik GmbH, and the results can be transferred to them.

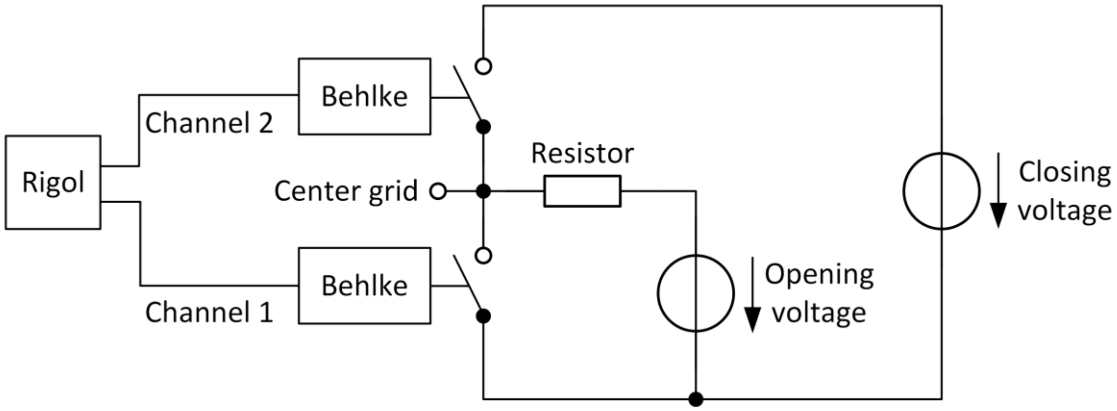

Figure S1: Example schematic diagram of the setup with commercially available components for providing the voltage sequence of the center grid of the tristate ion shutter. Using a two-channel Rigol DG4062 function generator two Behlke HTS31-06-C high voltage switches and two ESC-2 XP Power isolated high voltage power supplies.

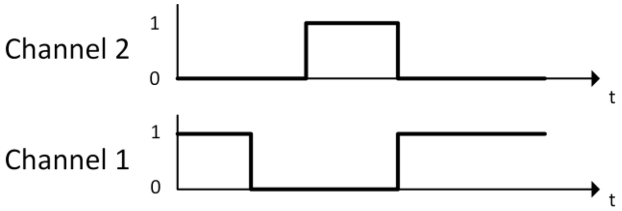

Figure S2: Pulse sequences of a two-channel function generator to exemplarily provide the voltage sequence of the middle grating of the tristate ion shutter.

Optimized Resolving Power

Shutter Opening Time

Video S1: COMSOL simulation of an ion injection in the Tristate shutter at extremely low opening times. The color gradient indicates ion density, while the white lines indicate the electrical field lines. The potential on the first (left) and third (right) grid remains constant, while the center grid is switched as detailed in Figure 2 panels e) to i) of the original publication[1]. However, instead of maintaining an open state where the potential on the center grid that lies between the potentials of the first and third grid, the potential of the center grid is switched directly from one closed state to the other. In other words, the opening time is practically zero and ions are only injected due to the field inhomogeneities present

Figure S3 shows the ion mobility spectra for 5 mg/l isoproturon and pyrimethanil, respectively, in 80:20 MeOH:H_2O for different ion shutter opening times. If the application requires the detection of the lowest possible concentrations, a higher ion shutter opening time should be selected. If the IMS provides a second separation dimension, a lower ion shutter opening time is recommended in order to maximize the resolving power.

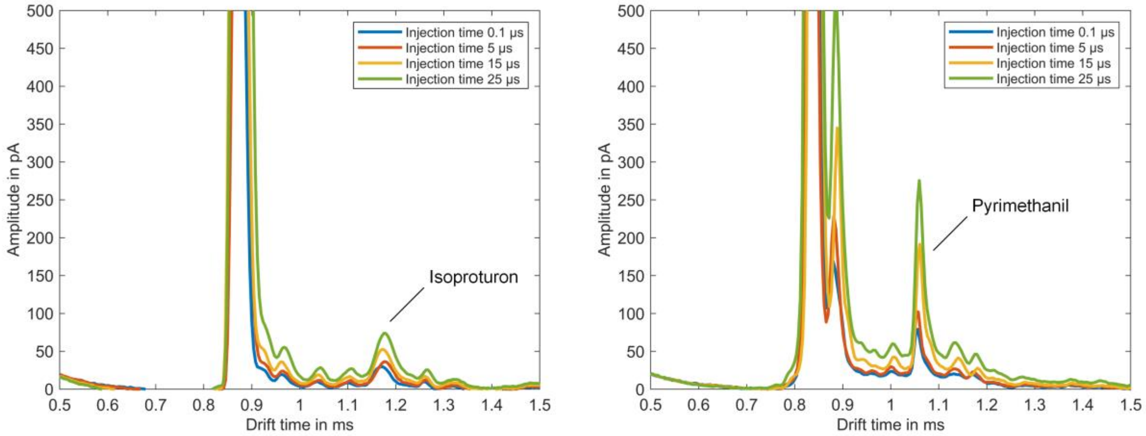

Figure S3. Ion mobility spectra of 5 mg/l isoproturon in 80:20 MeOH:H_2O (left) and of 5 mg/l pyrimethanil in 80:20 MeOH:H_2O (right) at a drift voltage of 7500 V for different injection times from 0.1 µs to 25 µs.

Transimpedance Amplifier

Figure S4 shows significant differences in the resolving power and amplitude of the respective analyte peaks for the different amplifiers. The two amplifiers with high bandwidths both provide a resolving power of $R_P = 92$ for the THAB peak, showing that excessive bandwidth does not provide any improvement of the ion mobility spectra with respect to resolving power. The amplifier with lower bandwidth of 29 kHz reduces resolving power down to $R_P = 77$, as the amplifier broadens the peak significantly.

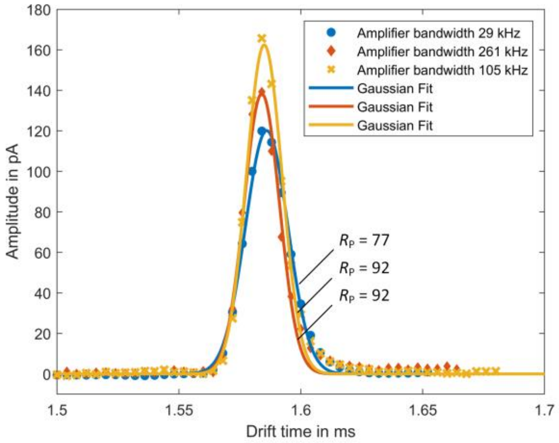

Figure S4. THAB peaks (extracted from the ion mobility spectra when electrospraying the four instrument standards TEAI, TPAI, TBAI and THAB dissolved n methanol) at a drift voltage of 7500 V when using a transimpedance amplifier with a bandwidth of 29 kHz (data points: blue circles, Gaussian fit: blue line), 261 kHz (data points: red diamond, Gaussian fit: red line) and 105 kHz (data points: yellow crosses, Gaussian fit: yellow line) and the corresponding resolving powers.

REFERENCES

(1) Kirk, A. T.; Grube, D.; Kobelt, T.; Wendt, C.; Zimmermann, S. A High Resolution High Kinetic Energy Ion Mobility Spectrometer Based on a Low-Discrimination Tristate Ion Shutter. *Anal. Chem.* **2018**, *90*, 5603-5611, DOI: 10.1021/acs.analchem.7b04586.
